# Glucocorticoid receptor is involved in the breed-dependent transcriptional regulation of 3β-hydroxysteroid dehydrogenase in the liver of preweaning piglets

**DOI:** 10.1186/s12917-015-0441-6

**Published:** 2015-05-26

**Authors:** Xian Li, Yimin Jia, Runsheng Li, Zhiyuan Sun, Xi Li, Shiyan Sui, Ruqian Zhao

**Affiliations:** Key Laboratory of Animal Physiology and Biochemistry, Nanjing Agricultural University, Nanjing, 210095 P. R. China

**Keywords:** AR, C/EBPβ, GR, Liver, Pig, 3β-HSD

## Abstract

**Background:**

Hepatic 3β-hydroxysteroid dehydrogenase (3β-HSD) plays an important role in steroid inactivation and catabolism. Serum concentrations of steroid hormones differ significantly between breeds in pigs, however the molecular mechanism regulating hepatic 3β-HSD expression in different breeds of pigs is poorly understood. In the present study, we used preweaning purebred male Large White (LW) and Erhualian (EHL) piglets as model to investigate the breed difference in the expression and regulation of *3β-HSD* gene in porcine liver.

**Results:**

The hepatic expression of *3β-HSD* mRNA was significantly lower (*P* < 0.01) in EHL piglets compared to that in LW piglets. Significant breed differences were detected for the hepatic expression of transcription factors such as androgen receptor (AR), glucocorticoid receptor (GR), and CCAAT/enhancer binding protein β (C/EBPβ). The nucleoprotein contents of AR (*P* < 0.05), GR (*P* < 0.01) and phospho-Ser^211^GR (*P* < 0.01) were significantly higher in the liver of EHL piglets. Chromatin immunoprecipitation (ChIP) assay demonstrated significantly lower binding of GR, but not AR or C/EBPβ, to 3β-HSD gene promoter in EHL piglets (*P* < 0.05). GR was not detected to interact with C/EBPβ or AR in the co-immunoprecipitation analysis.

**Conclusions:**

These results indicate that GR binding to *3β-HSD* promoter is involved in the breed-dependent *3β-HSD* expression in the liver of piglets.

## Background

The enzyme 3β-hydroxysteroid dehydrogenase (3β-HSD) is widely distributed in various tissues of animals. In steroidogenic tissues such as adrenal gland and gonads, 3β-HSD is essential for the biosynthesis of steroid hormones [1, 2]. In peripheral tissues such as liver and kidney, 3β-HSD catalyzes the conversion of steroids from active to inactive forms [2, 3]. Liver is the main site for steroids inactivation and catabolism [4–7]. Boar taint, an unpleasant odor of cooked pork or pork products, is partly contributed by the high level of androstenone in pig adipose tissue [8]. Boar taint can be reduced by castration, which removes the potential for androstenone production. However, due to animal welfare issues and decreasing in production efficiency caused by castration, elimination of boar taint through alternative methods would be of great benefit to the pork industry. In porcine liver, 3β-HSD is responsible for the initial step of androstenone metabolism [9–12]. Pigs with low hepatic 3β-HSD expression and activity showed a reduced rate of hepatic androstenone clearance and the accumulation of androstenone in adipose tissue [9, 13–15].

The expression of *3β-HSD* is predominantly regulated at the level of transcription [2, 16]. A wide array of transcription factors have been identified to participate in the transcriptional regulation of *3β-HSD* gene [17–20]. For instance, CCAAT/enhancer binding protein β (C/EBPβ), a member of CCAAT/enhancer binding protein family, has been reported to participate in the breed-specific expression of adrenal *3β-HSD* in pigs [21]. Steroid hormones are involved in the modulation of 3β-HSD expression [2, 16]. Androgens have been reported to inhibit 3β-HSD expression in Leydig cells [22, 23] and adrenal fasciculata cells [24], whereas glucocorticoids stimulate 3β-HSD expression in human adrenocortical NCI-H295R cells [25] but inhibit 3β-HSD expression and activity in rat Leydig cells [26]. To date, majority of the studies has been focusing on the 3β-HSD gene regulation in steroidogenic tissues, little is known about the transcriptional regulation of 3β-HSD gene in the liver.

Chinese native pig breeds have been reported to have significantly higher plasma cortisol and testosterone concentrations compared to the Western pig breeds [21, 27–29]. Moreover, Chinese Erhualian (EHL) pigs were found to express higher glucocorticoid receptor (*GR*) mRNA in the liver compared to Pietrain pigs [30]. In our previous study, we found that GR and androgen receptor (AR) participated in the regulation of 3β-HSD expression via protein-protein interaction with C/EBPβ in procine adrenal glands [21]. However, it remains unknown whether breed differences in plasma cortisol and testosterone concentrations are associated with breed disparities in hepatic *3β-HSD* expression, and whether GR and AR are involved in the breed-dependent transcriptional regulation of *3β-HSD* in porcine liver.

Therefore, in the present study, purebred EHL and Large White (LW) preweaning male piglets with different concentrations of serum cortisol and testosterone were employed as model to investigate the breed differences in hepatic 3β-HSD expression and to explore the possible mechanisms underlying the breed-dependent transcriptional regulation of *3β-HSD* gene in the liver, including the role of transcription factors AR, GR and C/EBPβ.

## Methods

### Animals

Five preweaning purebred male LW (body weight, 8.10 ± 0.34 kg) and 6 EHL (body weight, 4.08 ± 0.25 kg) piglets at the age of 25 days (d) were respectively obtained from two neighboring pig breeding farms in Changzhou, Jiangsu Province, China. As large animals, 5 to 6 pigs are enough for the lab experiments. The piglets were exsanguinated after electric stunning by a licensed slaughterhouse staff on the respective farm. Liver samples from the same regions were frozen in liquid nitrogen immediately after collection and then stored at −70 °C for further analysis. The Animal Ethics Committee at Nanjing Agricultural University reviewed the protocol and approved this study specifically, with the project number 2009ZX08009-138B. The slaughter and sampling procedures complied with the “Guidelines on Ethical Treatment of Experimental Animals” (2006) No. 398 set by the Ministry of Science and Technology, China and “the Regulation regarding the Management and Treatment of Experimental Animals” (2008) No.45 set by the Jiangsu Provincial People’s Government.

### RNA isolation, cDNA synthesis and real-time PCR

Total RNA was isolated from liver samples using Trizol Reagent (Invitrogen, USA) and then purified with DNase I (RNase Free, D2215, Takara, Japan) according to the manufacturer’s instructions. Concentration of the extracted RNA was measured using NanoDrop 1000 Spectrophotometer. Denaturing agarose electrophoresis was used to confirm RNA integrity. Real-time PCR was carried out to examine DNA contamination. M-MLV reverse transcriptase (Promega, Madison, WI, USA) were used to synthesize cDNA with 2 μg of total RNA following the manufacturer’s instructions. Two microlitres of diluted cDNA (1:50) were used for real-time PCR using SYBR Green Real-time PCR Master Mix (TaKaRa, Japan) in Mx3000P (Stratagene, USA). All primers (Table 1) were synthesized by Generay Biotech Co., Ltd. (Shanghai, China). Several reference genes (*18S*, *GAPDH*, *ACTB* and *PPIA*) were tested for mRNA quantification. The expression stability was evaluated by geNorm [31] and peptidylprolyl isomerase A (*PPIA*) was shown to be the most stable and did not show difference in expression between indigenous Chinese pig breeds and western breeds [32]. Therefore *PPIA* was chosen as the reference gene. The method of 2^-ΔΔCt^ was used to analyze the real-time PCR data [33], and the mRNA levels were expressed as the fold change relative to the mean value of LW pigs.

**Table 1:** Primers used in the present study

Name	Sequence	Application
3β-HSD (NM_001004049.1)	F: TTCCTGGCAAGTATTTCTCGG	mRNA expression
	R: TCCAGCAACAAGTGGACGAT	
GR (AY779185)	F: CCAAACTCTGCCTTGTGTGTTC	
	R: TGTGCTGTCCTTCCACTGCT	
C/EBPβ (NM_001199889.1)	F: GACAAGCACAGCGACGAGTA	
	R: AGCTGCTCCACCTTCTTCTG	
AR (NM_214314.2)	F: AGCACCATACGGCTACAC	
	R: GCCCATCTCGCTTTTGAC	
PPIA (NM_214353.1)	F: GACTGAGTGGTTGGATGG	
	R: TGATCTTCTTGCTGGTCTT	
C/EBPβ-Site1 (−2078/-1953)	F: ACTGCGGAGGGGCTATTG	ChIP for 3β-HSD promoter AR, GR and C/EBPβ binding
	R: CTCAGAACTCATGCGACCAG	
C/EBPβ-Site2/3 GR (−723/-576)	F: TGAGACTTTGGCCGCAATC	
	R: GTGACTTGTGGCCTTATGCA	
C/EBPβ-Site4 GR (−45/+171) AR (−45/+171)	F: GCTGAGTCAGAGGCAAGGG	
	R: GAGGTCAGGTGATTCAGTGGAA	
GR (−371/-178)	F: TAATCCACTGGGCGAGAC	
	R: AACCACAGCAGGAGAAAGAT	
AR (−298/-79)	F: CCATGACAGGGACTCCTCC	
	R: AGCTCACGCCTCCTTACTTC	
AR (−5279/-4985)	F: CCTCAAGGATAGAAGGATGG	
	R: CAATGGGGAAAAGCGTAG	

### Protein extraction and Western blot analysis

Nuclearprotein extracts from 100 mg frozen liver tissue were prepared as previously described with some modifications [34]. Briefly, approximately 100 mg frozen liver samples were homogenized in 1 mL of low-salt buffer (20 mM HEPES pH 8.0, 10 mM KCl, 1 mM EDTA, 1 mM EGTA, 0.2 % NP-40, 10 % glycerol) containing protease inhibitors cocktail (05892791001, Roche, Germany) and phosphatase inhibitors cocktail (4906837001, Roche, Germany), and incubated for 10 min on ice. After centrifugation for 1 min at 13,000 g at 4 °C, the remaining pellet was dissolved in 100 μL high-salt buffer (420 mM NaCl, 20 mM HEPES pH 8.0, 10 mM KCl, 1 mM EDTA, 1 mM EGTA, 20 % glycerol, protease inhibitor cocktail, phosphatase inhibitors cocktail), kept on ice for 30 min, and then centrifuged for 10 min at 13,000 g at 4 °C. Supernatants were taken as nuclear fractions. Protein concentration was measured with the BCA Protein Assay Kit (Pierce, Rockford, IL, USA) according to the manufacturer’s instructions. After denaturation, 40 μg of protein, which was calibrated as the optimum loading quantity, was electrophoresed in a 7.5 % or 10 % SDS-PAGE gel together with a protein size ladder (SM0671, Fermentas), and the separated proteins were transferred onto the nitrocellulose membranes (BioTrace, Pall Co., USA). Western blot analysis for GR (sc-1004×, Santa Cruz, USA, diluted 1:2000), phospho-Ser^211^GR (ab55189, Abcam, USA, diluted 1:1000), AR (sc-815×, Santa Cruz, USA, diluted 1:2000) and C/EBPβ (sc-150×, Santa Cruz, USA, diluted 1:2000) was carried out according to the recommended protocols provided by the manufacturers. Histone H1 (BS1655, bioworld, USA, diluted 1:500) was used as a reference. Finally, the blots were detected by enhanced chemiluminescence (ECL) using the LumiGlo substrate (Super Signal West Pico Trial Kit, Pierce, USA). The ECL signals were recorded by the VERSADOC MP 4000 system (Bio-Rad, USA) and analyzed with Quantity One software (Bio-Rad, USA).

The GR, phospho-Ser^211^GR, AR, C/EBPβ and Histone H1 antibodies worked well in the detection of porcine proteins in high specificity, and the main band appeared at the expected molecular weight. So we cut the gel for these antibodies to include all 11 samples in one blot. The Western blot results were repeated twice.

### Transcription factors’ prediction and Chromatin immunoprecipitation (ChIP)

The glucocorticoid responsive element (GRE), androgen responsive element (ARE) and C/EBPβ binding sites were predicted on pig 3β-HSD gene promoter, by using TF Search (http://www.cbrc.jp/research/db/TFSEARCH.html) and gene-regulation (http://www.gene-regulation.com/pub/programs.html) online resources.

ChIP analysis was performed according to our previous publication [21, 35]. In brief, 100 mg frozen liver samples were ground in liquid nitrogen and resuspended in PBS. After cross-linking in 1 % formaldehyde, the reaction was stopped using 2.5 M glycine. The pellets were washed using PBS and lysed with SDS lysis buffer containing protease inhibitors cocktail. The chromatin was sonicated on ice to yield DNA fragments of 200 to 500 bp in length. Then the protein-DNA complex was diluted in ChIP dilution buffer containing protease inhibitors. After pre-clearance of the chromatin preparations with salmon sperm DNA/ protein A/G plus beads (40 μL, 50 % slurry, sc-2003, Santa Cruz, CA, USA), the immunoprecipitation was performed with 4 μg GR (sc-1004×, Santa Cruz, USA), AR (sc-815×, Santa Cruz, USA), C/EBPβ (sc-150×, Santa Cruz, USA) and normal rabbit IgG (sc-2027, Santa Cruz, USA) (negative control) overnight at 4 °C, respectively. Protein A/G plus beads (40 μL, 50 % slurry) were added to capture the immunoprecipitated chromatin complexes for 2 h at 4 °C. Finally, reverse cross-linking was performed to release DNA fragments from the immunoprecipitated complex at 65 °C for overnight and DNA was purified. Immunoprecipitated DNA was used as template for real-time PCR and specific primers were designed to amplify genomic sequences at the promoter region of 3β-HSD gene (Table 1). ChIP results were normalized to the rabbit normal IgG precipitation and shown as fold enrichment.

### Co-immunoprecipitation

Co-immunoprecipitation was performed as previously described with minor modifications [36]. Five hundred micrograms of total protein were precleared with 40 μL of protein A/G plus beads for 1 h at 4 °C, then centrifuged at 1,000 × g for 5 min. The supernatants were incubated with 4 μg C/EBPβ and GR antibodies and rotated overnight at 4 °C. Thereafter, 40 μL of agarose beeds were incubated with the protein-antibody complexes for 2 h at 4 °C. After centrifugation, the agarose beeds were washed and the immunoprecipitated proteins were run on 7.5 % SDS-polyacrylamide gel for western blot analysis.

### Statistical analysis

All data were presented as mean ± SEM, and were analyzed using independent-samples *t-*test with SPSS 21.0 for Windows. The mRNA or protein expression was expressed as the fold change relative to LW. Difference was considered significant when *P* < 0.05.

## Results

### Hepatic expression of 3β-HSD gene

**Fig. 1:**
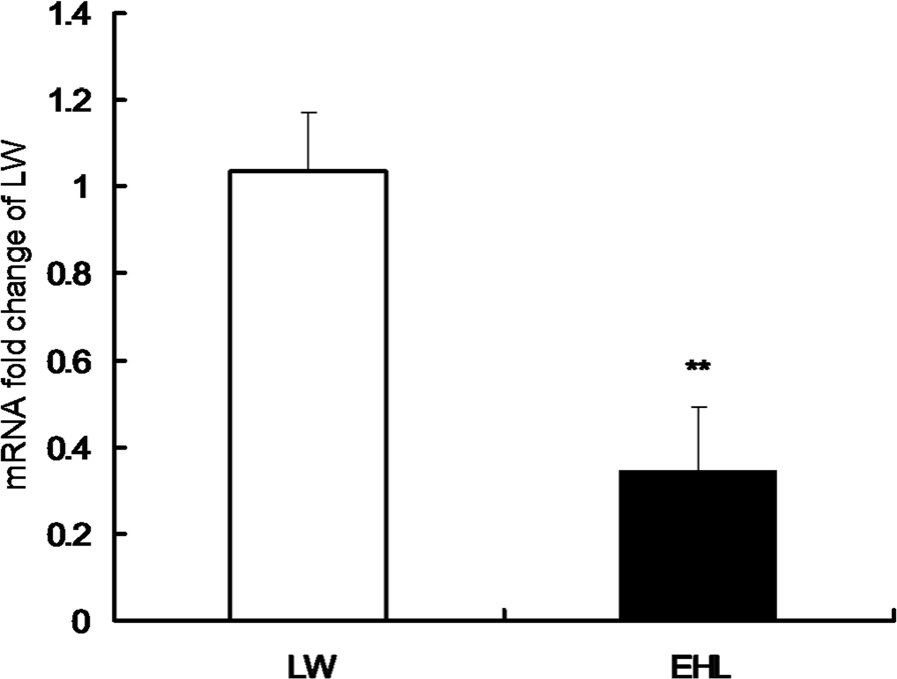
Hepatic expression of porcine 3β-HSD gene. Values are means ± SEM, n = 5 for LW or 6 for EHL. ** P < 0.01, * P < 0.05, compared with LW

EHL piglets showed significantly lower hepatic *3β-HSD* mRNA expression as compared to LW piglets (*P* < 0.01) (Fig. 1).

### Hepatic expression of GR, AR and C/EBPβ

**Fig. 2:**
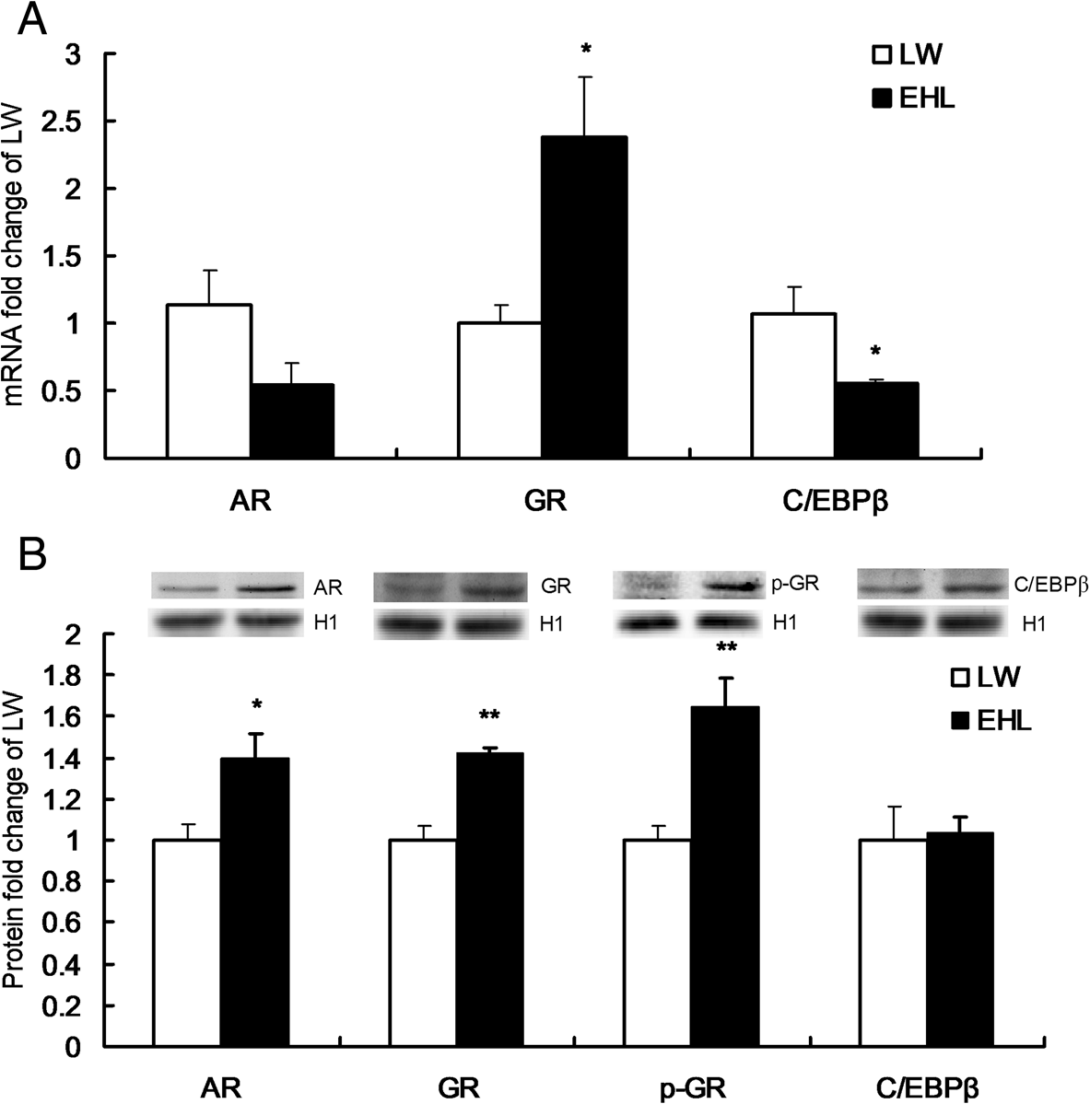
Relative Hepatic expression of transcription factors. (**a**) Hepatic expression of genes involved in 3β-HSD transcription. (**b**) Nucleoprotein content of AR, GR, phospho-Ser211GR and C/EBPβ in the liver. Values are means ± SEM, n = 5 for LW or 6 for EHL. * P < 0.05, compared with LW

The mRNA expression of *AR* tended to be lower in the liver of EHL piglets compared to that in LW (*P* = 0.07) (Fig. 2a). However, the hepatic nucleoprotein content of AR was significantly higher in EHL piglets (*P* < 0.05) (Fig. 2b). Similarly, the hepatic mRNA expression and nucleoprotein contents of GR (*P* < 0.01) and phospho-Ser^211^GR (*P* < 0.01) were significantly higher in EHL piglets (Fig. 2a and b). *C/EBPβ* mRNA expression was significantly lower in the liver of EHL piglets (*P* < 0.05), but no breed difference was observed for the protein content (Fig. 2a and b).

### Binding of GR, AR and C/EBPβ to 3β-HSD promoter

**Fig. 3:**
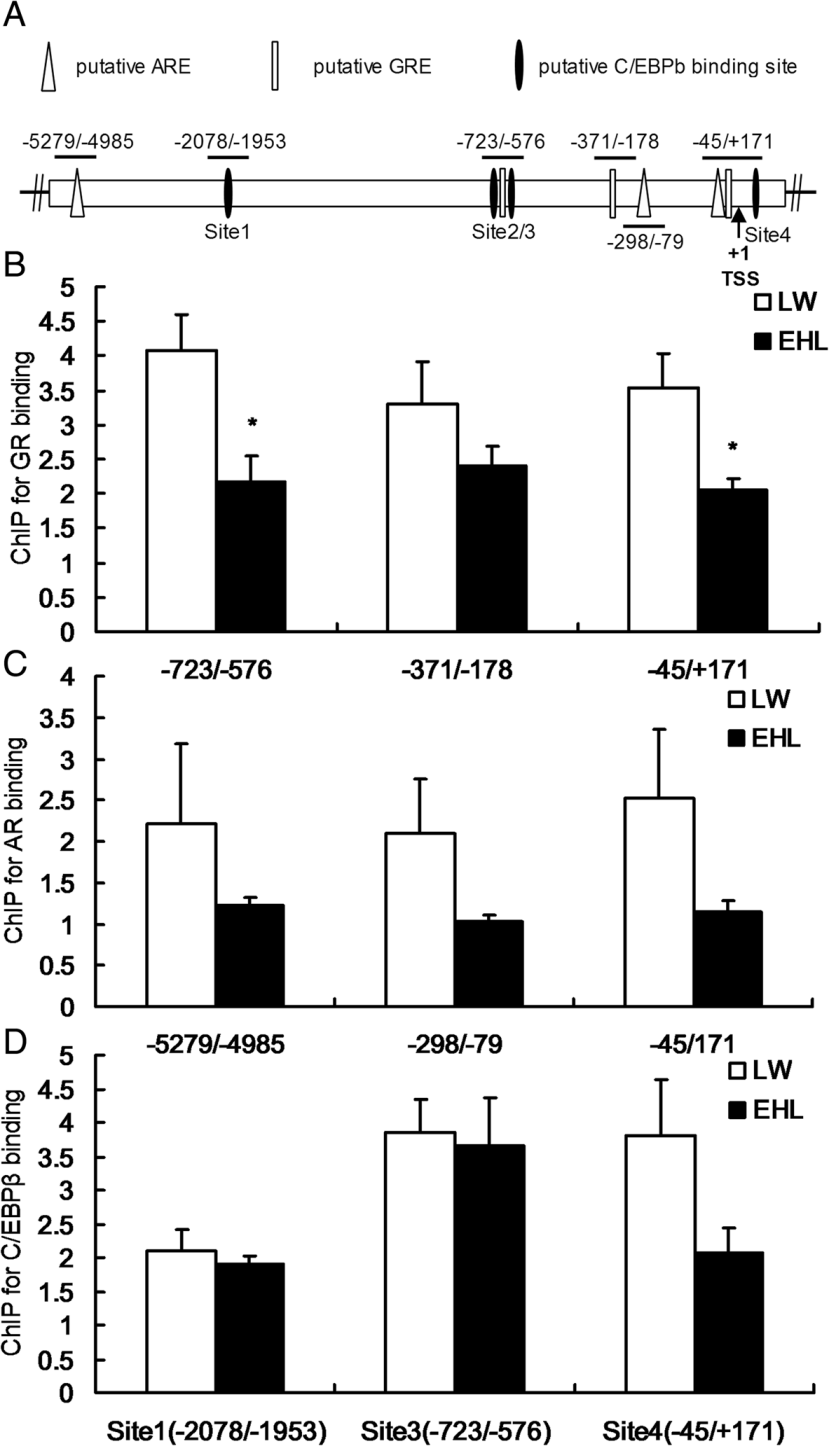
ChIP analysis for GR, AR and C/EBPβ binding on hepatic 3β-HSD promoter. (**a**) Schematic structure of porcine 3β-HSD promoter. GRE, ARE and C/EBPβ binding sites and the PCR fragments used to detect GR, AR and C/EBPβ binding are indicated; TSS: transcription start site. (**b**) GR binding on hepatic 3β-HSD promoter. (**c**) AR binding on hepatic 3β-HSD promoter. (**d**) C/EBPβ binding on hepatic 3β-HSD promoter. Values are means ± SEM, n = 5 for LW or 6 for EHL. * P < 0.05, compared with LW

Three putative GREs, 3 AREs and 4 C/EBPβ binding sites were predicted on pig 3β-HSD gene promoter (Fig. 3a). ChIP assay revealed significantly lower binding of GR to the first and the third predicted GREs on 3β-HSD promoter in the liver of EHL piglets (*P* < 0.05, Fig. 3b). No significant breed difference was observed for the binding of AR or C/EBPβ to 3β-HSD promoter in the liver of preweaning piglets (Fig. 3c and d).

### Interaction between GR and C/EBPβ or AR

**Fig. 4:**
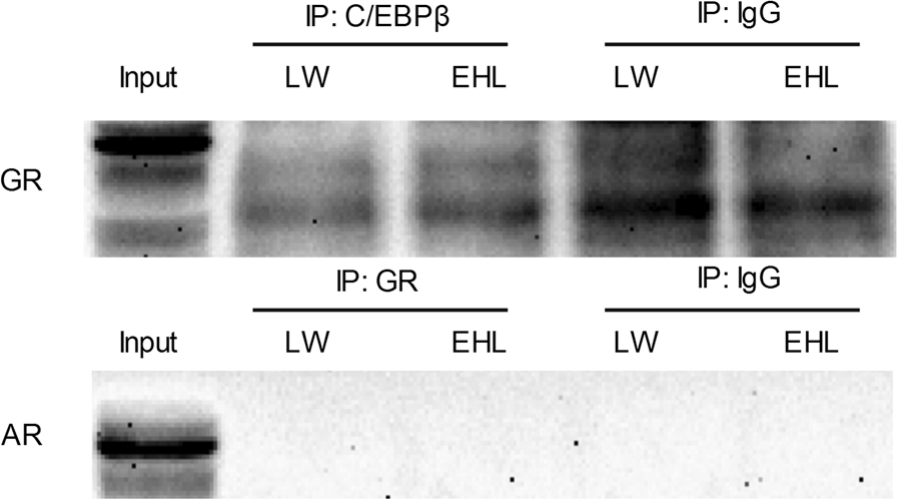
Western blot detection of GR or AR protein, after co-immunoprecipitation with C/EBPβ or GR antibody. The pooled samples of 5 LW pigs and 6 EHL pigs were used in the detection. The input lane in detection of the pooled LW sample is shown as marker. IP, immunoprecipitate

Co-immunoprecipitation analysis revealed that neither AR nor C/EBPβ was detected to interact with GR in the liver of EHL and LW piglets (Fig. 4).

## Discussion

In this study, we provide the first evidence on the breed-dependent regulation of hepatic *3β-HSD* gene transcription in the pig. EHL piglets expressed significantly lower *3β-HSD* mRNA in the liver as compared with LW piglets. This is in line with a previous report that the expression of *3β-HSD* mRNA was about 12 times higher in the liver of crossbred LW pigs compared to that in crossbred Meishan pigs [9]. However, the result is contrary to our previous study in adrenal gland, in which EHL piglets expressed significantly higher *3β-HSD* mRNA compared to LW piglets [21]. It suggests that different regulatory mechanisms exist in liver and adrenal gland about *3β-HSD* expression.

Steroid hormones such as cortisol and testosterone may affect the transcriptional regulation of *3β-HSD* through their respective nuclear receptors. Previously, we found that GR and AR participated in the regulation of *3β-HSD* expression via protein-protein interaction with C/EBPβ in procine adrenal glands [21]. In the present study, *GR* mRNA abundance and the nuclear content of phospho-Ser^211^GR were significantly higher in the liver of EHL piglets as compared to that in LW piglets. This is in agreement with our previous finding that EHL pigs express higher abundance of *GR* mRNA in the liver as compared to Pietrain pigs [30]. The relationship between glucocorticoid concentration and GR expression is complex and appears to be species-specific. For instance, a synthetic glucocorticoid dexamethasone was reported to inhibit hepatic *GR* mRNA and protein levels in rats [37] and mice [38], while in Italian chickens, the hepatic GR expression was negatively correlated with the blood corticosterone level [39]. In contrast to GR, AR mRNA and protein expression seems to be uncoupled. EHL piglets were detected lower *AR* mRNA (*P* = 0.07) yet higher AR protein in the liver as compared with LW piglets. In the process of gene transcription, protein or even phosphorylated protein levels play the pivotal regulatory roles [40]. The inconsistence between mRNA and protein suggests a post-transcriptional regulation. A similar phenomenon was described previously that testosterone decreases *AR* mRNA but increases AR protein in human prostate adenocarcinoma (LNCaP) cells [41].

We reported previously that C/EBPβ is involved in the breed-specific expression of 3β-HSD in porcine adrenal glands [21]. In this study, *C/EBPβ* mRNA expression was down-regulated in the liver of EHL piglets, but no breed difference was detected for the C/EBPβ protein content. It happens that the mRNA abundance of a gene and its protein content do not match, indicating post-transcriptional or translational regulation. We did not examine the level of phosphorylated C/EBPβ. So it is unknown whether breed differences can be detected for phosphorylated of C/EBPβ. It is reported that C/EBPβ [42–44] and AR [45] can regulate the transcription of target genes via protein-protein interactions with GR. Using online transcription factors prediction databases, we located several putative GREs, AREs and C/EBPβ binding sites on the proximal promoter sequence of porcine *3β-HSD* gene. ChIP analysis revealed significant breed difference in the GR binding to the two predicted GREs on *3β-HSD* promoter, with lower binding detected in the liver of EHL piglets. This finding implicates the direct involvement of GR in the transcriptional regulation of porcine *3β-HSD* gene in the liver. ChIP assay is used for detecting the binding of a transcription factor with its response elements on gene promoters or regulatory sequences in tissue or cell lysates under real biological situation. Compared with electrophoretic mobility shift assay which uses labelled synthetic oligonucleotide probe to bind a transcription factor in nuclear extracts in vitro, ChIP assay reflects the binding status under real tissue or cell conditions. Certainly, binding itself is not linked directly to the function of transcritional activation or repression. A functional promoter activity analysis is required to evaluate the function. However, promoter activity analysis using a dual-luciferase reporter system has been also performed under an artificial scenario using transfected cell models in vitro. We did not have a porcine hepatocyte cell line and failed to transfect the primary porcine hepatocytes for functional promoter activity analysis. It is surprising that higher GR nuclear translocation in the liver of EHL piglets coincided with lower GR binding to the *3β-HSD* gene promoter. Nevertheless, the uncoupling of nuclear protein content and GR binding to a target gene promoter is not an unique observation. It was reported that *Mycoplasma hyopneumoniae* infection promotes GR-mediated toll-like receptor 2 activation by increasing GR binding, without affecting GR nuclear translocation in porcine lung [46]. To elucidate whether AR and C/EBPβ also contribute to the breed difference in hepatic *3β-HSD* gene transcription in porcine liver, we also detected binding of these two transcriptional factors to *3β-HSD* gene promoter and their interactions with GR. It appears that AR and C/EBPβ are unlikely to contribute to the breed disparity in hepatic *3β-HSD* expression in EHL and LW piglets through either direct binding to the promoter, or indirect protein-protein interaction with GR.

## Conclusions

Taken together, we show that the hepatic expression of *3β-HSD* gene is regulated differently between LW and EHL piglets, at the level of transcription. The GR binding to 3β-HSD promoter is responsible, at least in part, for the breed-dependent *3β-HSD* expression in liver of piglets.

## Abbreviations

3β-HSD: 3β-hydroxysteroid dehydrogenase
AR: Androgen receptor
ARE: Androgen responsive element
C/EBPβ: CCAAT/enhancer binding protein β
ChIP: Chromatin immunoprecipitation
EHL: Erhualian
GR: Glucocorticoid receptor
GRE: Glucocorticoid responsive element
LNCaP: Human prostate adenocarcinoma
LW: Large white
PPIA: Peptidylprolyl isomerase A
